# Barriers and facilitators to the provision of optimal obstetric and neonatal emergency care and to the implementation of simulation-enhanced mentorship in primary care facilities in Bihar, India: a qualitative study

**DOI:** 10.1186/s12884-018-2059-8

**Published:** 2018-10-25

**Authors:** Melissa C. Morgan, Jessica Dyer, Aranzazu Abril, Amelia Christmas, Tanmay Mahapatra, Aritra Das, Dilys M. Walker

**Affiliations:** 1 Department of Pediatrics, University of California San Francisco, 550 16th Street, Box 1224, San Francisco, CA 94158 USA; 2 Institute for Global Health Sciences, University of California San Francisco, 550 16th Street, Box 1224, San Francisco, CA 94158 USA; 3 Maternal, Adolescent, Reproductive, and Child Health Centre, London School of Hygiene & Tropical Medicine, Keppel Street, London, WC1E 7HT UK; 4 Pronto International, 5419 Greenwood Avenue North, Seattle, WA 98103 USA; 5 Médecins Sans Frontières, Nou de la Rambla 26, 08001 Barcelona, Spain; 6 Pronto International; State RMNCH+A Unit, C-16 Krishi Nagar, A.G. Colony, Patna, Bihar 80002 India; 7 CARE India, 14 Patliputra Colony, Patna, Bihar 800013 India; 8 Department of Obstetrics, Gynecology, and Reproductive Sciences, University of California San Francisco, 1001 Potrero Ave, San Francisco, CA 94110 USA

**Keywords:** Nurse mentoring, Nurse mentorship, Obstetric care, Neonatal care, Rural health, Bihar, India

## Abstract

**Background:**

Globally, an estimated 275,000 maternal deaths, 2.7 million neonatal deaths, and 2.6 million third trimester stillbirths occurred in 2015. Major improvements could be achieved by providing effective care in low- and middle-income countries, where the majority of these deaths occur. Mentoring programs have become a popular modality to improve knowledge and skills among providers in low-resource settings. Thus, a detailed understanding of interrelated factors affecting care provision and mentorship is necessary both to improve the quality of care and to maximize the impact of mentoring programs.

**Methods:**

In partnership with the Government of Bihar, CARE India and PRONTO International implemented simulation-enhanced mentoring in 320 primary health clinics (PHC) across the state of Bihar, India from 2015 to 2017, within the context of the AMANAT mobile nurse mentoring program. Between June and August 2016, we conducted semi-structured interviews with 20 AMANAT nurse mentors to explore barriers and facilitators to optimal care provision and to implementation of simulation-enhanced mentorship in PHCs in Bihar. Data were analyzed using the thematic content approach.

**Results:**

Mentors identified numerous factors affecting care provision and mentorship, many of which were interdependent. Such barriers included human resource shortages, nurse-nurse hierarchy, distance between labor and training rooms, cultural norms, and low skill level and resistance to change among mentees. In contrast, physical resource shortages, doctor-nurse hierarchy, corruption, and violence against providers posed barriers to care provision alone. Facilitators included improved skills and confidence among providers, inclusion of doctors in training, increased training frequency, establishment of strong mentor-mentee relationships, administrative support, and nursing supervision and feedback.

**Conclusions:**

This study has identified many interrelated factors affecting care provision and mentorship in Bihar. The mentoring program was not designed to address several barriers, including resource shortages, facility infrastructure, corruption, and cultural norms. These require government support, community awareness, and other systemic changes. Programs may be adapted to address some barriers beyond knowledge and skill deficiencies, notably hierarchy, violence against providers, and certain cultural taboos. An in-depth understanding of barriers and facilitators is essential to enable the design of targeted interventions to improve maternal and neonatal survival in Bihar and related contexts.

**Electronic supplementary material:**

The online version of this article (10.1186/s12884-018-2059-8) contains supplementary material, which is available to authorized users.

## Background

Globally, an estimated 275,000 maternal deaths, 2.7 million neonatal deaths, and 2.6 million third trimester stillbirths, defined as fetal death at ≥28 weeks of gestation, occurred in 2015 [1–3]. Direct obstetric causes accounted for 86% of maternal deaths, among which postpartum hemorrhage (PPH) was the principal cause [1]. Preterm birth complications (35%), intrapartum-related events (24%), and infection (15%) were the leading causes of neonatal death [2]. Risk of mortality is highest in labor and on the day of birth, during which time 46% of maternal deaths and stillbirths and 36% of neonatal deaths occur [4]. To help reduce preventable deaths during this critical period, the World Health Organization advocates for all births to be attended by a a skilled health provider [5]. Further, estimates suggest that trained midwives, who are regulated under international standards, have the competencies to provide 87% of essential maternal and newborn healthcare services [6].

In 2015, India alone accounted for 23% and 26% of global maternal and neonatal deaths, respectively [1, 2]. In an effort to improve survival among mothers and newborns, the Indian Government launched two initiatives to promote institutional deliveries across the country. Established in 2005, Janani Suraksha Yojana (JSY) provided cash incentives to pregnant women, facilitated by Accredited Social Health Activists (ASHA), to deliver in health facilities [7, 8]. In 2011, the Government of India started Janani Sishu Suraksha Karyakram (JSSK), which provides free delivery and neonatal care as well as transport to and from health facilities [9]. To maximize impact, these initiatives focused on states with relatively high rates of fertility and neonatal mortality, such as Bihar in northeastern India, which has also been found to be the poorest region in all of South Asia [10]. Between 2002 and 2016, the institutional delivery rate across India increased from 19% to 79% [11], and in Bihar, from 20% to 64% [12]. However, while the rate of institutional delivery has risen, the quality of care in facilities is often suboptimal and coverage inadequate, especially in rural areas [13–16].

In partnership with the Government of Bihar, CARE India implemented a mobile nurse mentoring program called AMANAT (Hindi for ‘emergency obstetric and neonatal readiness’) in 2012 [17]. Through this program, trained nurse mentors visited primary health clinics (PHC) in pairs, conducting week-long visits to four PHCs every month over a period of 7 to 8 months to train nurse midwives. In 2014, CARE India partnered with PRONTO International to incorporate simulation and team training into the AMANAT program [18]. PRONTO is a highly realistic, simulation-based obstetric and neonatal emergency training program designed for low-resource settings [19, 20]. Simulation-enhanced mentoring was implemented in 320 PHCs across Bihar between 2015 and 2017, as described in detail elsewhere [21].

Mentoring programs have become a popular training model to address deficiencies in knowledge and skills among skilled birth attendants, typically employing a combination of bedside teaching, didactic lectures, skills stations, and simulations. However, little is known about the obstacles and enablers of this training model. Further, essential maternal and neonatal health interventions are not successfully implemented in many low- and middle-income countries due to underlying constraints, particularly regarding workforce, financing, and service delivery [22]. An in-depth understanding of interrelated factors affecting care delivery and mentoring in such contexts is necessary both to improve the quality of care and to maximize the impact of mentoring programs. This study aimed to qualitatively explore barriers and facilitators to the provision of optimal obstetric and neonatal emergency care and to the implementation of simulation-enhanced mentorship in primary care facilities in Bihar.

## Methods

### Setting

Bihar has a population of over 100 million, of which 87% is rural [12]. According to the most recently available estimates, the maternal mortality ratio was 274 (2013) and the neonatal mortality rate was 37 (2016) [7, 12], compared to 248 and 25, respectively, for India as a whole [1, 23]. In Bihar, each PHC serves an average population of 45,000 [24]. Obstetric and newborn care at PHCs are provided by nurses with either an Auxiliary Nurse Midwifery (ANM) or a General Nursing and Midwifery (GNM) qualification, which entail two years and three and a half years of training, respectively, following completion of secondary school [25].

### Data Collection

We conducted semi-structured interviews with 20 nurse mentors, purposively selected from the total pool of 120 AMANAT nurse mentors who had provided simulation-enhanced mentoring. Participants were selected based on the following criteria: 1) nurse mentor employed by AMANAT at the time of interview, and 2) completed ≥1 phase of AMANAT (equivalent to 8 months). Participants were selected from intervention sites across 11 districts to gain maximum diversity and generate richer information (Additional file 1). Participants were recruited until thematic saturation was achieved [26]. Interviews were conducted between June and August 2016. Duration ranged from 40 to 60 minutes. The interview guide (Additional file 2) was developed in English, translated to Hindi, and translated back to English in a blinded manner to ensure accuracy and equivalence [27]. Interviews were conducted in the language of the participant’s preference, either English or Hindi. Interviews were conducted by a co-author (AA) and an Indian research assistant. Both were females who had received training in qualitative research skills. Two pilot interviews were conducted to identify unclear interview questions, allowing refinement of the interview guide. These were not included in the final analysis. Questions were open-ended to allow participants to expand upon topics they felt were important, allowing the interviewer to ask new questions on emerging themes. Interviews were held in private rooms at PHCs to ensure anonymity and confidentiality.

### Data Analysis

Interviews were transcribed and, where necessary, translated to English by three Indian research assistants. Transcriptions were done following the True Verbatim method to accurately capture meanings, perceptions, and context [28]. To ensure data quality, two independent staff double-checked all transcriptions and translations. Data were analyzed using the thematic content approach [26], consisting of four steps: 1) familiarization; 2) identifying codes and themes; 3) coding the data; 4) organizing codes and themes. The thematic content approach is broadly used in qualitative research and aims to present the main elements of the participants’ descriptions [26]. The first author (MM) and one interviewer (AA) read all transcripts and developed the preliminary coding scheme together. Two interviews were double-coded by the first author and a co-author (JD). Any inconsistencies were discussed and resolved to develop the final coding framework. The first author coded all remaining interviews. New themes, which could not be placed within the established coding framework, were also included [29, 30]. The consolidated criteria for reporting qualitative research [31] guided reporting for this study (Additional file 3).

## Results

We interviewed 20 nurse mentors. Table 1 shows the participant characteristics.

**Table 1:** Participant characteristics (*N*=20)

Characteristic	*n* (%)
Female	20 (100)
Age, years (median, range)	24 (22-33)
Time working as mentor, months (median, range)	12 (9-18)
State of birth	
Delhi	7 (35)
West Bengal	4 (20)
Kerala	3 (15)
Maharashtra	2 (10)
Bihar	2 (10)
Other (Odisha, Uttar Pradesh)	2 (10)
Previous clinical experience	
Nursing school only	10 (50)
Worked as staff nurse	10 (50)
Previous teaching experience	
None	16 (80)
Nursing school tutor	2 (10)
Other (lecturer, nurse educator)	2 (10)
Primary reason for becoming a nurse mentor	
Gain teaching experience	9 (45)
Gain clinical experience	5 (25)
Wanted to work in Bihar	4 (20)
Salary	2 (10)
Career plans after this mentoring phase	
Continue mentoring in AMANAT program	14 (70)
Pursue master’s degree	3 (15)
Apply for different mentoring program	1 (5)
Apply for clinical nursing position	1 (5)

We use the main themes emerging from the data to structure the presentation of material from the interviews, broadly classified as barriers (Table 2) and facilitators (Table 3).

**Table 2:** Barriers to care provision and mentorship

Theme	Area(s) affected
Physical resources	Care provision
Facility layout	Care provision, mentorship
Human resources	Care provision, mentorship
Doctor-nurse hierarchy	Care provision
Nurse-nurse hierarchy	Care provision, mentorship
Corruption and fear	Care provision
Cultural issues	Care provision, mentorship
Low baseline skill level	Care provision, mentorship
Resistance to change	Mentorship

**Table 3:** Facilitators of care provision and mentorship

Theme	Area(s) affected
Improved skills and confidence through training	Care provision, mentorship
Refresher training, increased training frequency	Care provision, mentorship
Establishment of strong mentor-mentee relationships	Care provision, mentorship
Administrative support	Care provision
Nursing supervision and feedback	Care provision

### Barriers

#### Physical resources (care provision)

Over three-quarters of participants cited lack of physical resources, including supplies and equipment, as a barrier to care provision. Resources that were often unavailable or non-functional included autoclaves (for sterilization), gloves, labor tables, intravenous catheters, and suction bulbs.

*“Here [beginning of the program], they are not using any instruments and they are conducting deliveries even without gloves.”* (Age 24, 12 months mentoring)

Several participants described persistent shortages of uterotonics, antibiotics, intravenous fluids, and antihypertensive agents. When medications were out of stock, patients or their family members were asked to purchase medications from outside pharmacies or, in emergency cases, nurses sometimes purchased medications for patients.

*“Once there was a patient with severe pre-eclampsia… We used to ask them to make something available, but they used to not make it available at all… Her BP was very high, so what could we do? We had to give her nifedipine. We had magnesium sulfate in that place, but what to do about nifedipine? We did not have nifedipine there. We can’t leave the patient, as her BP is so high… and how can we refer her with this high BP? We had to reduce her BP, so we went to get nifedipine from the outside.”* (Age 22, 9 months mentoring)

*“Drugs were not at all available, so we ourselves have to go to store, tell the MOIC [Medical Officer In-Charge] about the drugs which are missing, medicines which are missing… We have to go and speak because the nurses would say, ‘Sister, we are fed up of telling them again and again, nothing is happening.’”* (Age 23, 9 months mentoring)

#### Facility layout (care provision and mentorship)

Mentors stated that distance between labor rooms and training rooms, where simulations were often conducted, was a common barrier to timely provision of emergency care and to mentorship.

*“Our training room... it was far from the labor room... we had to see the patient and also the training too... we did not get to know when a patient came in full dilatation and her delivery was done in the ward... ASHA came and shouted at us so much, saying, ‘Did you come here to see patients or to kill them? Patient’s delivery is happening in the ward and you all are not seeing it.”* (Age 22, 9 months mentoring)

#### Human resources (care provision and mentorship)

One-quarter of participants described staffing shortages as a significant barrier to both care provision and mentorship. They explained it was common in PHCs for a single nurse to cover the outpatient department, immunization duties, emergency room, and labor room. Further, participants stated that heavy delivery loads made it difficult to find time to conduct simulations. In addition, several explained that mentees were frequently assigned to night duty before or after training, leading to fatigue with a negative effect on work performance.

#### Doctor-nurse hierarchy (care provision)

Nearly three-quarters of participants stated that many doctors in PHCs failed to follow evidence-based care guidelines, citing this a major barrier to care provision. Further, doctors frequently argued with nurses who attempted to follow such guidelines.

*“I think they [mentees] are not... trusting in our mean doctors... In complication management, what we are telling them [mentees], doctors are telling some other things... so they are in conflict to listen to us or the doctor.”* (Age 24, 10 months mentoring)

*“’Sir [speaking to doctor], this is... PPH is happening, so we have to give’... He [doctor] will tell, ‘No, no, give dexamethasone’... We told him, ‘Actually sir, let’s not give dexamethasone... What if we start RL [Ringer’s lactate] today... as it has been given in the guidelines that we should do all these steps.’ Then he [doctor] told, ‘You don’t have to teach us... we know how we should manage.’”* (Age 22, 9 months mentoring)

Almost half of participants stated that doctors were unwilling to treat complications, instead instructing nurses to immediately refer such patients. Several participants felt this was due to doctors lacking of a sense of responsibility for patient care.

*“If there is a complication, they [male doctors] will not see the patient. They will ask the sister and then just order... ‘Ok, you want this medication to be written, I’ll write this’… attitude is there. And if there is a female [doctor]... she will be getting so much irritated… ‘Why are you calling every time? This is not normal process, natural process. You have 35 years’ experience, do nicely.’… They are fighting with sisters like that.”* (Age 22, 18 months mentoring)

*“Some MOICs and some doctors have even told us in a way like, if the mentees face any problem, then refer the patient as fast as possible. If the patient dies, then it will be out of our boundary that the patient will die.”* (Age 23, 10 months mentoring)

*“If something happens here, the public will tell me only that, ‘You did not take care well’… Doctors also, even before we do anything, keep telling, ‘Refer the patient, refer the patient’… like this it happens here, so I feel that doctors should be more involved in patients’ care.”* (Age 26, 18 months mentoring)

Over one-third of participants described doctors refusing to conduct rounds or see patients. Others stated that doctors were very slow to come even in emergency situations; for example, taking 10 to 15 minutes to attend to an asphyxiated newborn requiring resuscitation.

*“They don’t even touch the patient... because they get paid even without touching... nobody is there to tell them... Already they are given a big position and considered in a high position... So why would they go out of the way and do hard work without any government pressure.”* (Age 23, 10 months mentoring)

*“[Doctors] don’t have any tension... [my] first time in Bihar, I saw two gynecologists posted in one hospital. But in that hospital also, if we are calling other after PPH... the doctor is coming after one and a half hours, where the person is going to be just collapsed.”* (Age 22, 18 months mentoring)

Several participants felt that some doctors were rude and disrespectful toward nurses.

*“Some doctors we have fought so much... They are making fun of us in public. They are not respecting us sometimes... we have a fight with many doctors.”* (Age 22, 18 months mentoring)

*“‘You can’t refer instead of me, you are not doctor... You can’t refer because you are only [a] mentor in this facility.’ Like that he was directly blaming me.”* (Age 23, 18 months mentoring)

#### Nurse-nurse hierarchy (care provision and mentorship)

Participants described the age gap between mentors and staff in PHCs as a significant barrier to mentorship. Over half felt that older mentees commonly perceived younger mentors as lacking experience and, as a result, were resistant to learning from them. Notably, 80% of participants had no prior teaching experience and 50% had recently graduated from nursing school (Table 1).

*“She [mentee] told me that, ‘I have 23 years’ experience, you were not born [at] that time I started working, so don’t teach me!’”* (Age 23, 11 months mentoring)

*“Like this age gap... they [mentees] used to not accept us, they used to cross question us... In starting in one of our PHCs... [mentee said] ‘These are like kids only, what they will teach us?’ But later on, when we started interacting with them... they were telling, ‘You really are teaching us new things we didn’t know.’”* (Age 24, 12 months mentoring)

Several participants also discussed barriers to care provision related to seniority level and nursing qualifications. In particular, nurses with GNM degrees tended to have less respect for those with ANM degrees. As a result, some mentees were not able to practice the skills they had learned.

*“We had a mentee who was really intelligent and she was of young age and she had a will to learn, and she learned all but she was not able to do her work... If she starts doing it [communication techniques], obviously, everyone will start praising her, so her senior will not allow to do her work.”* (Age 24, 9 months mentoring)

#### Corruption (care provision)

Half of participants described various forms of corruption affecting patient care. Several stated that nurses collected money directly from patients for conducting deliveries. As a result, senior nurses often did not allow junior nurses to conduct deliveries. Participants explained that administrators commonly condoned this practice because they received a portion of these incentives.

*“It’s the same ego problem, even if we tell them... in 3 weeks, you have to do this much deliveries, then they would take us to the side sometimes and say that the one who has duty, they only do, they do not let us do it... there is some issue with money, like that who does the delivery gets some money.”* (Age 22, 12 months mentoring)

*“If I say that, ‘We should talk to the manager that your mentee is doing like this.’ I would like to tell you that the manager is also taking money from someone, incentive from someone, so why will he stop the money... some percentage is divided, so why will they [laughs], you know, spoil their incentives.”(Age 22, 18 months mentoring)*

In addition, participants described that it was common for nurses to run private clinics in their own homes to make extra money. Nurses with private clinics often prioritized these activities over their duties in the respective PHC, and many would refer patients with complications to their clinics instead of to the nearest district hospital. A monetary incentive was typically provided to doctors and MOICs to allow these practices to take place.

*“It’s very common... everybody knows this.... they themselves would tell their rate, for normal delivery we take 5000, for complicated delivery we take 8000. In fact, some mentees were so... I don’t know... should I call them greedy?... There is a complication... she would say, ‘Go to my clinic, I will come.’ After the training, she would go to her clinic, and we would come to know the complication, which we had referred [to the district hospital], happened in her clinic.”*(Age 23, 10 months mentoring)

Further, participants described that ASHAs received financial incentives to discourage patients from going to district hospitals and to instead bring patients to nurses’ private clinics. They stated that some ASHAs conducted vaginal exams to earn extra money, despite lack of training and being warned against this practice by supervisors in PHCs.

*“ASHA used to say sometimes that, ‘No, these people are doing like this. We go to DH [district hospital], so much problems occur, it is very far and it is expensive also. So, it is better it happens here. We’ll conduct your delivery’... they will tell like that... they will go to private practice like the nurses are doing now... they will tell like this... ‘You will go to there, you will spend so much money- 5000 directly... We’ll get 4000, 3000, and we’ll do everything.’”*(Age 22, 9 months mentoring)

*“If the ASHA does PV [per vaginal] from home and comes, they would get money for it... They were not doing it properly and that was the problem... sometimes… after doing PV only, the ASHA used to bring... so that they did not have to wait for long time, they will bring after full dilatation, will get the delivery done... they will get the money and also the patient.”*(Age 22, 9 months mentoring)

One participant explained that some administrators instructed providers not to record complications occurring in PHCs. Another described how some staff working in PHCs sold government-issued equipment and commodities for personal monetary gain.

*“We’ll ask them to write all the complications... but when we go after three weeks, not many complications would have occurred... Then we came to know through the nurses that MOIC and BHM [Block Health Manager] had forced them, saying, ‘Don’t write any complication. We don’t want any complication.’”* (Age 22, 9 months mentoring)

*“Some persons from the PHCs are getting many things from the government, they are getting many equipments, many things, but they are selling in the private areas. They are selling and getting more money from that.” (Age 22, 18 months mentoring)*

#### Fear (care provision)

Several participants stated that nurses in PHCs feared being blamed by doctors or authorities or being punished by patients’ families if something goes wrong. As a result, nurses often refused to manage complicated patients, instead referring them to another facility.

*“[Before simulation training] mentees would not even enter the delivery room... because of fear that they won’t be able to do it alone or what would happen… ‘If anything goes wrong then the public and the doctor would beat us.’”* (Age 26, 18 months mentoring)

*“They are afraid in some at the higher authorities... and in some local peoples... they are fearing... because if anything happens to the patient, they kill that nurse... In Bihar, many PHCs [are] like that... If complicated cases come, they are not managed.”* (Age 33, 18 months mentoring)

#### Cultural issues (care provision and mentorship)

Participants described an overall lack of awareness about the value of medical care during childbirth in Bihar. In addition, misconceptions surrounding the use of intrauterine contraceptive devices (IUCD) were prevalent in local communities.

*“People from Bihar itself, not from anywhere... they are saying like, ‘Everyone, dogs, cows are giving birth so, yes, human beings are also giving birth, there is not much to think so much to have a tension so much... We don’t need all these things.”* (Age 22, 18 months mentoring)

*“Even if one [woman with IUCD] has bleeding, now then, the whole village would come to know and it would become taboo… that IUCD would lead to bleeding, then cancer, this and that, even now people say the same thing, ‘No, no, no, we don’t want to put anything inside... cancer would happen’... We would give counseling you know, we would concentrate much on pre-counseling, post-counseling, then we made it as a routine after patient delivered, but still that taboo… exactly because this thing was spread in their village, that it would lead to cancer, there would be more bleeding, they can’t have child, can’t have boy child, womb would turn.”* (Age 23, 10 months mentoring)

In addition, participants stated that preference for male children was common in Bihar. This led to neglect of female newborns, with some families threatening or even abusing nurses who attempted to resuscitate them.

*“We have got abused while resuscitating a female baby… ‘Why do you want to give life to her?... Before we have three baby, four baby, all the four are females, and now which delivery took place, even that is female... These people who have come from outside- because of them, this happened... That sister, if she would have conducted the delivery, we would have got a male’... So, we faced all this.”* (Age 23, 10 months mentoring)

Participants also discussed several cultural barriers to simulation-enhanced mentorship. They explained that, among local communities, belief that male doctors are not allowed in the labor room was widespread. As a result, some mentees felt ashamed or uncomfortable acting as patients in simulations when male staff were present. For religious reasons, some mentees refused to lie on the labor table during simulations and others were reluctant to enter the labor room. One participant described a situation where a mentee had psychosomatic symptoms after acting as the patient in a PPH simulation, which was perceived as a bad omen by fellow mentees.

*“Sometimes they have rituals… Once, one mentee… it was a PPH scenario and there was bleeding, it was the artificial blood. After going home, she started having some problems like headache and knee pain… she became very scared that, ‘No, today in the PPH [simulation], I bled so much. Because of that, I had so much problem at home.’ So, all the mentees started having this thought… that this is a bad omen. With lots of difficulty we explained to them… after that, for two days, we only acted as patients then we told them, ‘Sister, it is nothing, you see, do and see. Nothing happened to us.’”* (Age 24, 9 months mentoring)

#### Low baseline skill level (care provision and mentorship)

Participants explained that low baseline skill level among mentees, coupled with longstanding use of non-evidence-based practices, posed barriers to both care provision and mentorship.

*“Yes, actually really it’s very bad things. The practices they are using... to initiate cry in baby... They are applying oil and tapping.”* (Age 24, 18 months mentoring)

*“The problem is with the malpractices they follow from the last 20 years... they have learned some wrong practices, and society has given them a good name.”* (Age 24, 9 months mentoring)

#### Resistance to change (mentorship)

Finally, participants described how resistance to change made mentorship challenging, especially at the beginning. In some cases, mentees refused to participate in training sessions due to arrogance and disinterest.

*“One of our facilities... it was one of our worst facilities actually... because the mentees, the administration, all were very rigid... rigid as in their behavior, in the system they didn’t want to change. In everything, there was an excuse.”* (Age 23, 10 months mentoring)

*“[Mentees] would be sitting around saying, ‘It is not our duty hours, so why should we work?’ At the beginning, they used to think like that only… Some of them did not change at all... like, ‘I know everything, I don’t need to learn.’”* (Age 22, 9 months mentoring)

### Facilitators

#### Improved skills and confidence through training (care provision and mentorship)

All participants agreed that training and mentorship improved providers’ skills and confidence, particularly with regard to managing common obstetric and neonatal complications, and that this was a key facilitator of evidence-based care provision and successful mentorship.

*“The first time they [mentees] were going [into the] labor room... they will say, ‘I can’t do delivery... never I would I have been doing,’ and now they are calling us, they are managing PPH, they are managing pre-eclampsia, they are managing eclampsia, they are managing birth asphyxia.”* (Age 22, 18 months mentoring)

*“A lot of changes have occurred in their knowledge and their practices. Earlier, in the beginning, mentees used to be a lot behind, they used to feel scared that, ‘We can’t do this, we can’t do this’... I mean they were scared to talk to MOIC... ‘Sir, how will we refer this patient?’ or ‘How will we do?’... they started doing everything by themselves very well… everything!”* (Age 22, 9 months mentoring)

*“[Previously] they are not bothering about the patient is bleeding or baby is not crying, anything they are not taking it serious[ly] because they are telling, ‘It’s luck, it’s her luck or the baby’s luck, he is going, he is not alive, so what to do with them’... But after that, we have seen that they have managed... complications about birth asphyxia, meconium or PPH.”* (Age 24, 10 months mentoring)

All participants agreed that simulation was a valuable aspect of training, particularly for teaching mentees how to manage complications seen less frequently in PHCs, such as shoulder dystocia and pre-eclampsia. The majority of participants felt that simulation was more effective than other training methods; however, they stressed the importance of providing lectures and skills stations to improve understanding of new content areas prior to conducting simulations in those areas.

*“Simulations… I think this is a very great idea... teaching somebody who has experience of 25 to 30 years and now we have to change his practice… After doing simulation, they’ll [mentees] learn how we are doing and what else we can do... Because if we are teaching them like, ‘You have to do this, this, and this,’ they’ll not understand, they’ll not even do that thing. But after doing simulation, they’ll remember all we have done… so we have to do these things in real life also.*” (Age 23, 11 months mentoring)

*“I’ll frankly say, after the lunch, my mentees will sleep for classroom training... But when we do the simulation, they [say], ‘Oh my god! What scenario will she give? What scenario?’... their mind starts working.”* (Age 22, 18 months mentoring)

*“In college, we used to read from books and, by reading, we would learn something and forget something. But here practically when we did things… we understood it deep, as in what it is. I didn’t have to open [a] book and read… to learn why is it like this, practically we did and the concept got cleared… I think simulation is better than anything because theory, anything would not go in their mind.”* (Age 23, 9 months mentoring)

Nearly all participants felt that doctors should also participate in training in order to ensure they are aware of current, evidence-based guidelines for obstetric and neonatal care. One explained that doctors in some PHCs participated in a clinical guidelines workshop that was very helpful because, after the workshop, doctors started listening to the nurses and asking questions.

*“When we ask[ed] them something or had to refer, then they [doctors] used to tell by themselves that, ‘No, no, we should not do this.’ For example, like when there is an asphyxiated baby, they say, ‘There is no role for dexamethasone here’... And that oxytocin should not be given before. In the beginning… they used to order to give misoprostol and methergin after every delivery. After that, they came to know and they used to say, ‘No, no, there is no role of that and we should give only oxytocin.’”* (Age 22, 9 months mentoring)

In addition, several participants felt that nurses who are not participating in the mentoring program should be included in training when possible. Related suggestions included pairing mentees with non-mentee nurses during work shifts and incorporating a short training session for both at the beginning of the mentoring period to cover basic skills, such as measuring vital signs and using a partograph.

#### Refresher training and increased training frequency (care provision and mentorship)

One-quarter of participants recommended refresher training and increased training frequency to improve both care provision and mentorship, explaining that mentees commonly forgot what they had previously learned following the three-week gaps between monthly mentoring visits.

*“They [mentees] say, ‘Sister, if this... training is done again or revision is done, then we will completely remember it.’ So, I asked that [mentee], ‘What happened now?’ Then she said... ‘Sister, you have taught us pulse or anything, but if we do not know the basic then what do we do?’ One mentee even said that, ‘It was 30 years already and I still did not know how to measure BP [blood pressure].... I used to feel very ashamed, but due to this training, I learned many basic things that I used to feel shy about to do.’”* (Age 24, 9 months mentoring)

*“When we leave one facility and go to the other and go back to that facility after three weeks, then some changes happen again. If the gap is reduced, then it will be so much better... because some changes start occurring during the week, but after that the training gets completed, and then they forget those things in the three-week gap. Then again, after three weeks, we have to start from bringing about new changes.”* (Age 26, 18 months mentoring)

One mentor discussed use of unplanned follow-up visits during non-mentoring weeks to motivate mentees who were not practicing, additionally noting the value of employing a combination of strictness and kindness to promote behavior change.

*“We used to go see what is happening... We thought that no follow up was happening at night of the third week, so they used to go at any time be it night, morning, or evening and they got used to it that people will come for visit at any time and if something is not done, they would be scolded… We had to be strict with them because, with love, we could only change the behaviors... We decided that I would be strict one and sister the loving one... so that they could share their problem with her... and I am strict, so they would be a little scared… At least they should be scared of one and other one should be good.”* (Age 22, 9 months mentoring)

#### Establishment of strong mentor-mentee relationships (care provision and mentorship)

Almost half of participants discussed the value of establishing strong relationships, based upon mutual respect and trust, with mentees. Role modeling and companionship were also seen as key components in building such relationships.

*“When we were given the ToT [training of trainers], then we were told that we have to maintain good relationship no matter how because, if it is maintained, then only they would listen to you... In the first week, any of the days, we used to do cleaning, organizing, and seeing this, that the mentors are doing it even though they come and go in big cars, means in spite of being in a better position they are doing it, then we should also be doing it... We all used to eat together, feed each other, we never thought anything otherwise.”* (Age 22, 9 months mentoring)

Participants also described the importance of being approachable and promoting open communication with mentees.

*“They [mentees] used to call us ma’am, actually, in starting but later we told them no use of this ma’am and all because we are really a facilitator, not a teacher... so that whatever doubts and whatever they have, they can come to us.”* (Age 24, 12 months mentoring)

*“In the beginning, it took a lot of time to develop a relationship… then after that, so much more… They [mentees] started sharing everything with us like, ‘All these things are happening here’… and if there was any complication in the night, also, they used to call us freely that, ‘Sister, there is a case like this, what should we do?’”* (Age 22, 9 months mentoring)

#### Administrative support (care provision)

Nearly three-quarters of participants discussed the importance of administrative support for improved care provision, particularly with regard to repair of broken equipment and replenishment of out-of-stock supplies and medications. One mentor recommended assigning experienced administrators to cover PHCs whose staff had not yet received mentoring.

*“We used to tell our district manager to come in this QI [quality improvement] meeting, as the gaps were more... the district manager was also not taking things seriously... When the feedback started going, then everyone started taking things seriously... When the QI meeting was there, then the MOIC felt guilty that in his PHC things were not available.”* (Age 22, 9 months mentoring)

#### Nursing supervision and feedback (care provision)

Several participants discussed the importance of nursing supervision and provision of performance feedback by local governmental employees, particularly during non-mentoring weeks (when mentors were not present), to encourage mentees to practice newly learned skills.

*“They [mentees] will practice during the mentoring week, but during the non-mentoring week, they will not practice until their own superiors create pressure... One thing is there should be some pressure from the administrative level. That does not happen. So, I mean, only during the mentoring week, they learn well, they understand well at that time itself. But later when we come again, we had to revise the same chapter again.”* (Age 24, 9 months mentoring)

One participant suggested appointing the most skilled and knowledgeable mentee at each PHC to serve as leaders, supervising other mentees and providing feedback to program staff.

*“The best mentee should be made the leader, so that they can supervise and should report what is not going on... this can help in sustaining [the effects of training].”* (Age 26, 18 months mentoring)

## Discussion

This study has demonstrated that there are a wide variety of barriers and facilitators to the provision of high quality obstetric and neonatal emergency care and to the implementation of simulation-enhanced mentorship in PHCs in Bihar, many of which are interdependent. Many of the barriers were related to the broader systemic context, and thus not directly addressed by the mentoring program. Others were related to the working culture in PHCs and providers’ knowledge, skills, and practices, which may be affected by simulation-enhanced mentorship.

### Barriers

Shortages of physical and human resources, coupled with high patient volume in PHCs, posed significant barriers to both care provision and mentorship. Similar resource constraints have been reported globally [32, 33] and in Bihar [24, 34]. Despite establishment of 1,045 new institutions offering GNM courses and 1,362 new institutions offering ANM courses across India between 2009 and 2015 [35, 36], estimates suggested shortages of approximately 1.3 million GNMs (178%) and 670,000 ANMs (185%) in 2015 [37]. Improved utilization of ASHAs and Ayurvedic, Yoga and Naturopathy, Unani, Siddha and Homoeopathy doctors may help relieve this burden by providing basic healthcare services, particularly at the community level [38]. Barriers related to doctor-nurse and nurse-nurse hierarchy were also prevalent in PHCs. Problems with doctors stemmed from disrespect, disagreement regarding clinical guidelines, and poor sense of responsibility for patient care; whereas among nurses, differences in age, seniority, or nursing qualifications were commonly implicated. Other studies have similarly noted hierarchical issues affecting the quality of healthcare in India [39, 40]. In this program, the fact that the mentors were young, with limited clinical and teaching experience, was an important limitation to their ability to effectively mentor.

Various forms of corruption affecting care provision were identified, including side payments for doctors and nurses and financial incentives for ASHAs. Corruption in healthcare has been recognized as a common problem in India [41–43]. Several reports have acknowledged corruption, primarily in the form of providers demanding side payments from patients, as a barrier to the success of the JSY and JSSK programs [44–46]. Such practices not only have negative financial consequences for patients; they may also hinder appropriate management and referral of patients with complications. Fear of blame and physical assault in response to poor outcomes was another significant barrier to care provision, leading providers to frequently refer complicated cases. Violence against providers has been increasing globally, due to mistrust of the medical profession, corruption and lack of faith in the judicial system, rising cost of healthcare, and insufficient security in many facilities [47–51]. In India, additional causes include low health literacy, poor quality of service, and widespread belief that patients’ families may perpetrate violence with impunity [49–51]. These factors create an environment where providers are disinclined to communicate details about medical emergencies to patients and their families, leading to a sense of perceived neglect that may trigger violence. A training or mentorship program that focuses on empathy and effective communication with patients may help to address this systemic issue [49, 50]. The next iteration of the PRONTO simulation curriculum in Bihar has been adapted accordingly.

Lack of awareness about the value of medical care during childbirth was found to be common in Bihar. A related study similarly identified that prevalent perception of childbirth as a ‘natural event,’ which does not require healthcare, was a key factor impeding the uptake of JSY services in other high-focus states [46]. Preference for male infants was another cultural barrier, which frequently led to neglectful care of female newborns. Recent demographic data supports this finding, additionally determining that sex-selective abortion is common in Bihar [12]. Finally, this study identified widespread misconceptions regarding use of IUCDs, including beliefs that they cause irregular bleeding, cancer, and infertility. Similar findings have been reported elsewhere in India [52]. In 2016, the contraceptive prevalence rate in Bihar was 24%, compared to 54% nationally, and the rate of IUCD use was 0.5%, compared to 1.5% nationally [11, 12]. A study in Bihar found that lack of trained providers, community awareness, and accessibility to quality services were the main causes for low acceptance of IUCDs, and suggested that mobile family planning units with trained and skilled providers may promote use in remote areas [53].

### Facilitators

Improved skills and confidence, inclusion of doctors in training, and increased training frequency were seen as key facilitators of evidence-based care provision and successful mentorship. A study in India found that a 16-week training program for medical officers increased the proportion of trainees performing basic emergency obstetric care [54]. Comparable findings have also been seen in other programs focusing on obstetric and neonatal provider training in low-resource settings, including Making it Happen [55], Helping Babies Breathe (HBB) [56, 57], and the Dakshata Initiative [58]. Unlike AMANAT, these programs were much shorter in duration (1-2 days), utilized mannequins (as opposed to patient actors), and were generally conducted outside trainees’ usual workplaces. A study of HBB training in Tanzania found that the pass percentage for knowledge tests decreased from 89% to 69% eight weeks after initial training, improving to 90% following refresher training [56]. A more recent study of HBB in India and Kenya found that the successful completion rate for skills evaluations decreased from 99% to 81% seven months after initial training [57]. Similarly, participants in this study felt that knowledge and skills declined between monthly visits, and increased training frequency and refresher training were recommended. A nurse mentoring study in Uganda also noted the value of refresher training [59]. A study in Indonesia found that peer review and follow-up training led to enhanced consolidation of provider knowledge and skills [60].

Establishment of strong mentor-mentee relationships was identified as a key facilitator of mentorship, encouraging mutual respect, trust, and self-reflection among mentees. Role modeling, approachability, and open communication were seen as vital aspects of this relationship. A study in the U.S. similarly identified open communication and accessibility; mutual respect and trust; goals and challenges; passion and inspiration; caring personal relationship; knowledge exchange; independence and collaboration; and role modeling as central components of an effective mentoring relationship [61]. The aforementioned Ugandan study also noted the importance of role modeling, approachability, and establishing rapport [59]. As shown in this study, this can be challenging if there are significant gaps in age and experience between mentors and mentees.

Administrative support was an additional facilitator of care provision, particularly with regard to achieving sustainable improvements. Panda and Thakur similarly suggested that management practices, including decision making norms, knowledge, and experience of administrators, are critical to ensure the successful delivery of public health services in India [62]. Finally, nursing supervision and feedback were identified as additional facilitators of mentoring. In Tanzania, a study suggested identifying champion mentees at each site to receive additional and continued distance mentorship, who could provide refresher trainings and assist with follow-up [63]. Similarly, one participant in this study recommended appointing lead mentees at each site to supervise other nurses and provide programmatic feedback. Routine maternal and perinatal mortality audits, including consistent cause of death classification and use of best practice guidelines to monitor performance, have also been shown to promote improved care at the facility level in low-resource settings [64].

### Limitations

This study has several limitations. Participants had experience mentoring in PHCs during the AMANAT program, but had not served as primary providers in PHCs; thus, any preconceptions they had about Bihar could have introduced information bias. However, all participants had at least 9 months of mentoring experience in four or more PHCs. Further, two participants were from Bihar and their responses were not notably different compared to those from other states. Feedback regarding the mentoring program was not obtained from government officials or doctors working in PHCs; however, interviews were conducted with nurse mentees. These findings will be presented in a future manuscript. As the interviewers were members of the PRONTO team who were involved in training mentors, respondents may have provided answers to please the interviewers. To increase content validity, a local Hindi interviewer was present at all interviews and participants were ensured their responses were completely confidential in nature.

### Overall recommendations

Many of the identified obstacles were interdependent, affecting both care provision and mentorship, including those related to human resources, hierarchy, cultural beliefs, and provider practices and behaviors. These factors are particularly important to recognize and to address when possible, as they negatively affect both the quality of facility-based care and the impact of mentorship. Simulation-enhanced mentoring programs may be contextually tailored to address key barriers beyond deficiencies in providers’ knowledge and skills, notably hierarchical issues in facilities, violence against providers, and cultural taboos regarding provision of delivery care by male doctors. This suggests that simulation-enhanced mentoring is an appropriate training model to improve obstetric and neonatal care at primary care facilities in Bihar.

## Conclusions

This study has identified numerous barriers and enablers to the provision of obstetric and neonatal emergency care and to the implementation of simulation-enhanced mentorship in Bihar. The mentoring program was not designed to address some obstacles, including resource shortages, facility infrastructure, corruption, and cultural norms, as these require government support, community awareness, and other systemic changes. Mentoring programs may be adapted to address some aspects of care provision beyond provider knowledge and skills, notably hierarchy in facilities, violence against providers, and certain cultural taboos, while simultaneously building confidence in simulation training. An in-depth understanding of key barriers and facilitators is essential to enable the design of contextually-targeted interventions to improve survival among mothers and newborns in Bihar and elsewhere in India.

## Additional files

Additional file 1: Districts in Bihar where interviews were conducted. This map shows the 11 districts throughout the state of Bihar where interviews were conducted. Source of map: https://commons.wikimedia.org/wiki/File:Bihar_district_map.PNG. (TIFF 8968 kb)

Additional file 2: Interview guide. This document includes the questions used during interviews with participating nurse mentors. (DOCX 91 kb)

Additional file 3: Consolidated criteria for reporting qualitative research (COREQ): 32-item checklist. This document reports how this study addressed the items in the COREQ checklist. (DOCX 129 kb)

## Abbreviations

AMANAT: Aapathekalin Matritva Aevum Naavjat Sishu Tatparta (Hindi for ‘emergency obstetric and neonatal readiness’)
ANM: Auxiliary Nurse Midwifery (degree)
ASHA: Accredited Social Health Activist
BHM: Block Health Manager
BP: Blood pressure
GNM: General Nursing and Midwifery (degree)
HBB: Helping Babies Breathe
IUCD: Intrauterine contraceptive device
JSSK: Janani Sishu Suraksha Karyakram
JSY: Janani Suraksha Yojana
MOIC: Medical Officer In-Charge
PHC: Primary health clinic
PPH: Postpartum hemorrhage
QI: Quality improvement
ToT: Training of trainers
