# Supplementary figures and images for: Barriers and facilitators to the provision of optimal obstetric and neonatal emergency care and to the implementation of simulation-enhanced mentorship in primary care facilities in Bihar, India: a qualitative study
Source: BMC Pregnancy Childbirth. 2018 Oct 25;18:420. doi: 10.1186/s12884-018-2059-8 (PMC6202860; doi:10.1186/s12884-018-2059-8)

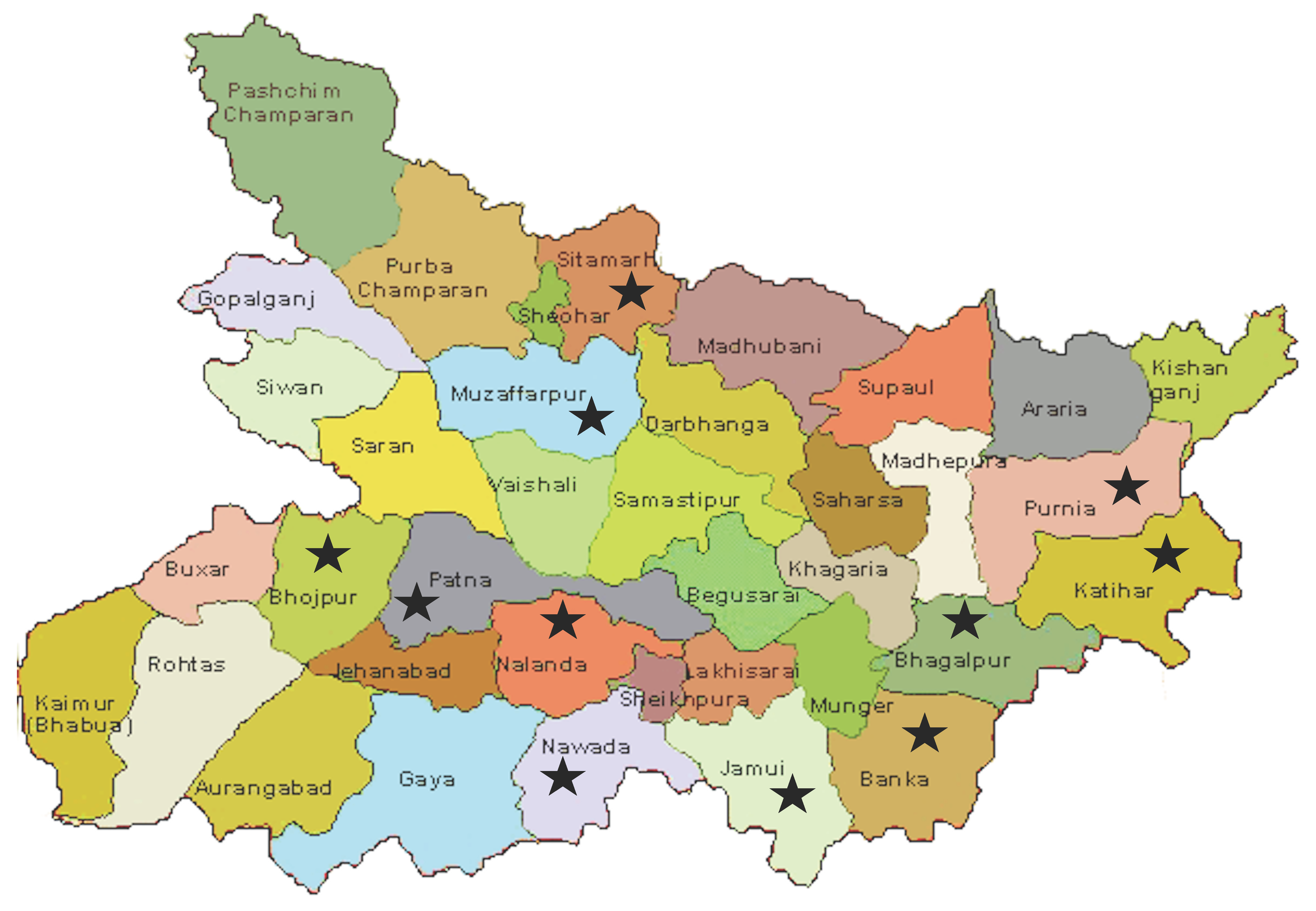

Supplement: Supplementary file 1 — Districts in Bihar where interviews were conducted. This map shows the 11 districts throughout the state of Bihar where interviews were conducted. Source of map: https://commons.wikimedia.org/wiki/File:Bihar_district_map.PNG. (TIFF 8968 kb) [file 12884_2018_2059_MOESM1_ESM.tiff]
