# Supplementary material for: Barriers and facilitators to the provision of optimal obstetric and neonatal emergency care and to the implementation of simulation-enhanced mentorship in primary care facilities in Bihar, India: a qualitative study
Source: BMC Pregnancy Childbirth. 2018 Oct 25;18:420. doi: 10.1186/s12884-018-2059-8 (PMC6202860; doi:10.1186/s12884-018-2059-8)
Supplement: Supplementary file 2 — Interview guide. This document includes the questions used during interviews with participating nurse mentors. (DOCX 91 kb) [file 12884_2018_2059_MOESM2_ESM.docx]

**Interview Guide – Nurse Mentors**

General questions

1. What is your age?
2. What state are you originally from?
3. For how many months have you been a nurse mentor?
4. How many years of nursing experience do you have?
5. What is your previous experience in training midwives? Please describe.
6. How did you come to learn about this mentoring program?
7. Why did you decide to participate in the mentoring program?
   1. Probe: incentives, altruism, other reasons?
8. Why do you think a mentoring program like this is needed in Bihar? Please describe.
9. In your words, what do you think the purpose of the mentoring program is?

Questions about simulation experiences

1. When you first learned about simulation training, what did you think?
2. When you were at a facility, how did you decide when to do a simulation?
3. How often did you do simulations at the facilities where you mentored?
   1. Probe: Do you think this was a good frequency?
   2. Probe: Would you have preferred more or fewer simulations?
4. Which were the topics the mentees understood best through simulation?
   1. Probe: Why do you think these topics were better understood?
5. Which were the topics the mentees did not understand in spite of simulation?
   1. Probe: Why do you think these topics were not understood?
6. How comfortable were your mentees in participating in simulations?
   1. Probe: What about older mentees?
7. How useful did you find simulation training for mentees? Please describe.
8. How did simulation fit with other methods of teaching, such as classroom lectures, skills stations, and practical/hands-on training that you used?
   1. Probe: Do you think the mentees could have benefited more from using other methods? Please, give examples.
   2. Probe: Compared to other methods, how did participating in simulations change how mentees cared for patients?
9. What challenges did you face in the use of simulation? Please describe.
   1. Probe: How did/do you deal with these challenges?
10. What do you think about debriefing?
    1. Probe: How useful is debriefing for mentees?
11. How do you decide what topics to include in debriefing?
12. What challenges did you face while debriefing?
    1. Probe How did/do you deal with these challenges?
13. What recommendations do you think can improve the use of simulation and debriefing to teach? Please describe.

Questions about mentoring experience

1. How has your mentoring experience been so far?
   1. Probe: Did you feel there was room for your suggestions to mentees?
2. How would you describe your relationship with your mentees?
   1. Probe: If your mentee was older and more experienced than you, how did you feel about teaching her? Was she open to your suggestions?
3. In your opinion, what do you think changed in the mentees’ attitudes and practices as a result of the mentoring program? Please describe.
   1. Probe: How has it changed mentees’ skills/confidence to provide patient care?
4. What challenges or problems did you encounter in the mentoring process? Please describe.
   1. Probe: religious beliefs, caste, support, supplies, other responsibilities?
   2. Probe: How did you address these problems?
5. What do you think about doctors’ role in patient care?
   1. Probe: Describe the current situation of how doctors care for patients.
   2. Probe: According to you, what should the role of the doctor be?
6. What suggestions do you have to improve the mentoring process? Please describe.
7. How can we sustain the changes that have been brought through the mentoring program?
8. What is your plan after the mentoring program?
