# Supplementary material for: Barriers and facilitators to the provision of optimal obstetric and neonatal emergency care and to the implementation of simulation-enhanced mentorship in primary care facilities in Bihar, India: a qualitative study
Source: BMC Pregnancy Childbirth. 2018 Oct 25;18:420. doi: 10.1186/s12884-018-2059-8 (PMC6202860; doi:10.1186/s12884-018-2059-8)
Supplement: Supplementary file 3 — Consolidated criteria for reporting qualitative research (COREQ): 32-item checklist. This document reports how this study addressed the items in the COREQ checklist. (DOCX 129 kb) [file 12884_2018_2059_MOESM3_ESM.docx]

**Consolidated criteria for reporting qualitative research (COREQ) checklist**

**Adapted from:** Tong A, Sainsbury P, Craig J. Consolidated criteria for reporting qualitative research (COREQ): a 32-item checklist for interviews and focus groups. Int J Qual Heal Care. 2007;19(6):349–57.

| \| **Number and item** \| \| --- \| | **Guide questions/responses** | **Section and page # where reported** |
| --- | --- | --- | --- |
| **Domain 1: Research team and reflexivity** | |  |
| *Personal Characteristics* | |  |
| 1. Interviewer | *Which author/s conducted the interviews?*  Interviews were conducted by AA and Praicey Thomas, a research assistant (see Acknowledgments). | Methods- data collection, paragraph 1 |
| 2. Credentials | *What were the researcher's credentials?*  Melissa Morgan, MD, MSc  Jessica Dyer, MPH  Aranzazu Abril, BA Midwifery (Hons), MSc Candidate  Amelia Christmas, MSc Tanmay Mahapatra, MBBS, PhD Aritra Das, MBBS, MS, PhD  Dilys Walker, MD | Title page |
| 3. Occupation | *What was their occupation at the time of the study?*  AA was a MSc student and PT was a Research Assistant. | N/A |
| 4. Gender | *Was the researcher male or female?*  Both interviewers were female. | Methods- data collection, paragraph 1 |
| 5. Experience and training | *What experience or training did the researcher have?*  MM had training in qualitative research methods during her MSc degree and has published research studies using qualitative methods. JD received training in and conducted qualitative research during her MPH degree. AA was a nurse midwife with experience instructing simulation trainings for midwives in Kenya, Senegal, South Sudan, and Iraq with Medecins Sans Frontieres. AA had previously assisted on other qualitative studies in the area of maternal health. AA and PT received formal training in qualitative research methods at the University of Surrey and the Public Health Foundation of India, respectively, in 2016. TM, AD, and DW are highly experienced researchers and have previously published studies using qualitative methods. | Interviewer training was reported: Methods- data collection, paragraph 1 |
| *Relationship with participants* | |  |
| 6. Relationship established | *Was a relationship established prior to study commencement?*  No prior relationship was established between interviewers and participants. | N/A |
| 7. Participant knowledge of the interviewer | *What did the participants know about the researcher?*  Reasons for conducting the study and institutional affiliations of all researchers were stated in the Participant Information Sheet and Consent Form. | N/A |
| 8. Interviewer characteristics | *What characteristics were reported about the interviewer?*  No interviewer characteristics were reported to participants. | N/A |
| **Domain 2: study design** | |  |
| *Theoretical framework* | |  |
| 9. Methodological orientation and theory | *What methodological orientation was stated to underpin the study?*  The thematic content approach was utilized. | Methods- data analysis, paragraph 1 |
| *Participant selection* | |  |
| 10. Sampling | *How were participants selected?*  Purposive sampling was utilized. | Methods- data collection, paragraph 1 |
| 11. Method of approach | *How were participants approached?*  Selected nurse mentors were approached face-to-face. | Methods- data collection, paragraph 1 |
| 12. Sample size | *How many participants were in the study?*  20 | Methods- data collection, paragraph 1 |
| 13. Non-participation | *How many people refused to participate or dropped out?*  No individuals refused to participate or dropped out of the study. | N/A |
| *Setting* | |  |
| 14. Setting of data collection | *Where was the data collected?*  All interviews were conducted at primary health clinics (PHC). | Methods- data collection, paragraph 1 |
| 15. Presence of non-participants | *Was anyone else present besides the participants and researchers?*  No, only the participant and interviewer were present. Interviews were held in private rooms at PHCs. | Methods- data collection, paragraph 1 |
| 16. Description of sample | *What are the important characteristics of the sample?*  Participants were females, aged 22-33 years, who had worked as nurse mentors in Bihar for 9-18 months prior to the time of the interview. Other important participant characteristics are shown in Table 1. | Results, paragraph 1 |
| *Data collection* | |  |
| 17. Interview guide | *Were questions, prompts, guides provided by the authors? Was it pilot tested?*  The interview guide was developed by the authors and prompts were given during interviews, if needed. Two pilot interviews were conducted. | Methods- data collection, paragraph 1 |
| 18. Repeat interviews | *Were repeat interviews carried out?*  No repeat interviews were conducted. | N/A |
| 19. Audio/visual recording | *Did the research use audio or visual recording to collect the data?*  All interviews were audio-recorded, with participants’ informed consent. | Declarations- consent for publication |
| 20. Field notes | *Were field notes made during and/or after the interview?*  Field notes were made to record additional information, as necessary. | N/A |
| 21. Duration | *What was the duration of the interviews?*  40-60 minutes | Methods- data collection, paragraph 1 |
| 22. Data saturation | *Was data saturation discussed?*  Yes, participants were recruited until thematic saturation was achieved. | Methods- data collection, paragraph 1 |
| 23. Transcripts returned | *Were transcripts returned to participants for comment and/or correction?*  No | N/A |
| **Domain 3: analysis and findings** | |  |
| *Data analysis* | |  |
| 24. Number of data coders | *How many data coders coded the data?*  Two interviews were double-coded by MM and JD. MM coded all remaining interviews. | Methods- data analysis, paragraph 1 |
| 25. Description of coding tree | *Did authors provide a description of the coding tree?*  Codes represented distinct viewpoints on each theme and subtheme. | N/A |
| 26. Derivation of themes | *Were themes identified in advance or derived from the data?*  Themes were derived from the data collected. | Methods- data analysis, paragraph 1 |
| 27. Software | *What software, if applicable, was used to manage the data?*  Transcripts were stored in Microsoft Word. | N/A |
| 28. Participant checking | *Did participants provide feedback on the findings?*  Participants did not provide feedback on the findings; however, findings will be shared with participants upon publication. | N/A |
| *Reporting* | |  |
| 29. Quotations presented | *Were participant quotations presented to illustrate the themes findings? Was each quotation identified?*  Yes, participant quotations were identified by age and duration of mentoring experience. | Results, all paragraphs |
| 30. Data and findings consistent | *Was there consistency between the data presented and the findings?*  Yes | Discussion, paragraphs 2-7 |
| 31. Clarity of major themes | *Were major themes clearly presented in the findings?*  Major themes resulting from the interviews are listed in Tables 2 and 3. | Results- Table 2 (barriers, paragraph 1); Table 3 (facilitators, paragraph 1) |
| 32. Clarity of minor themes | *Is there a description of diverse cases or discussion of minor themes?*  No, minor themes were not discussed. | N/A |
